# Association of Preoperative Cerebrospinal Fluids Parameters With Early Shunt Obstruction in Patients With Post-hemorrhagic Hydrocephalus Treated by Lumboperitoneal Shunt

**DOI:** 10.3389/fneur.2021.693554

**Published:** 2021-08-30

**Authors:** Tong Sun, Wenyao Cui, Siyang Chen, Yikai Yuan, Jingguo Yang, Yicheng Zhou, Xuepei Li, Hang Yu, Chao You, Junwen Guan

**Affiliations:** ^1^Department of Neurosurgery, West China Hospital, Sichuan University, Chengdu, China; ^2^Health Management Center, West China Fourth Hospital, Sichuan University, Chengdu, China; ^3^Medical Simulation Center, Chengdu First People's Hospital, Chengdu, China; ^4^Department of Neurology, Sichuan Provincial People's Hospital, University of Electronic Science and Technology of China, Chengdu, China; ^5^West China Brain Research Center, West China Hospital, Sichuan University, Chengdu, China

**Keywords:** lumboperitoneal shunt, post-hemorrhagic hydrocephalus, clinical outcomes, shunt obstruction, risk factor

## Abstract

**Background:** Early shunt obstruction (SO) remains the most common cause of lumboperitoneal shunt (LPS) failure. Although there is anecdotal evidence that the level of cerebrospinal fluid (CSF) parameters might affect shunt performance, its association with early LPS obstruction in adults with post-hemorrhagic hydrocephalus (PHH) is unclear.

**Methods:** The retrospective study was performed by reviewing the adults with PHH treated by LPS from years 2014 to 2018. We included patients with CSF samples analyzed within 1 week prior to shunt insertion or at the time of shunt insertion. Baseline characteristics of each patient were collected. The primary outcomes were the incidence rate and associated factors of SO occurring within 3 months of shunt placement. The secondary outcomes included scores on the National Institute of Health Stroke Scale (NIHSS) and Evans Index at discharge.

**Results:** A total of 76 eligible patients were analyzed, of whom 61 were obstruction-free and 15 were early SO. The overall rate of early SO was 15.6%. The RBCs count and nucleated cells count in preoperative CSF were actually higher in patients with early SO, compared to patients in the control group. Multivariate analysis identified RBC elevation (>0 × 10^6^/L; OR: 10.629, 95% CI: 1.238–91.224, *p* = 0.031) as a dependent risk factor for early SO. NIHSS dramatically decreased at discharge while the alteration of ventricular size was not observed.

**Conclusions:** This study suggested that the presence of RBCs in preoperative CSF was associated with early SO in patients with PHH treated by LPS.

## Introduction

Intraventricular hemorrhage (IVH) and subarachnoid hemorrhage (SAH) frequently lead to post-hemorrhagic hydrocephalus (PHH), resulting in ventriculomegaly, intracranial hypertension, and damage of the periventricular parenchyma (1). Cerebrospinal fluid (CSF) shunts, including ventriculoperitoneal shunt (VPS) and lumboperitoneal shunt (LPS), have long been used as the mainstay of PHH treatments (2). Specifically, VPS placement remains the most common used treatment while LPS serves as an effectively alternative option (3, 4). A growing number of neurosurgeons choose LPS placement for hydrocephalus, particularly in patients with idiopathic normal pressure hydrocephalus (INPH), since LPS has several advantages over VPS including the relatively low risk of brain injury and infection (5). In Japan, LPS has become the superior option in patients with INPH (6, 7). Besides, the indications for performing LPS have recently broadened to other communicating types of hydrocephalus, including PHH (8, 9). Wang et al. (10) recently found that patients with PHH treated by LPS or VPS obtained equal outcomes.

Despite immediate alleviation of symptoms after LPS placement, early shunt obstruction (SO) is unacceptable and is the most common cause of shunt failure (11, 12). The incidence rate of SO after primary shunt insertion is ~10.0% according to various studies, and SO frequently occurs within the first year (13, 14). Although there is anecdotal evidence that the level of CSF parameters might affect shunt performance, its association with early LPS obstruction in adult patients with PHH is unclear. This study aims to assess whether CSF parameters are associated with early SO following LPS implantation in a group of adult patients with PHH.

## Methods

### Patients

The retrospective study was performed by reviewing the adult patients with PHH treated by LPS at West China Hospital from years 2014 to 2018. We included patients with CSF samples analyzed within 1 week prior to shunt insertion or at the time of shunt insertion. Patients aged below 18 years, together with patients who were lost to follow-up, were excluded. Since bleeding at the time of lumbar puncture might distort the RBC count, we excluded patients with confirmed or susceptible bleeding that was related to lumbar puncture. LPS was totally performed by skilled and practiced neurosurgeons, and the shunting system with programmable pressure valve was obtained from either Medtronic (USA) or Sophysa (France).

### Baseline Characteristics

Baseline characteristics of each patient including age, sex, duration of hydrocephalus, history of prior shunt, follow-up time, symptoms, National Institute of Health stroke scale (NIHSS), Glasgow Coma Scale (GCS), and brand of shunt system were collected. CSF parameters including red blood cell (RBC) count, nucleated cell count, protein level, glucose level, and chlorine level were analyzed within 1 week prior to shunt insertion or at the time of shunt implantation.

### Outcomes

The primary outcomes were the incidence rate and associated factors of SO occurring within 3 months of shunt placement. Based on a previous study, obstruction was defined as a blockage of the catheter with debris, such as blood, proteinaceous fluid, or pieces of choroid plexus, which was confirmed within the process of shunt revision (13). Patients with an improvement of at least one point on the scores of NIHSS and without any shunt failure were included in the “obstruction-free” group (control group). The secondary outcomes included NIHSS and Evans Index at the time of discharge, corresponding to the improvement of symptoms and ventricular size. Additionally, the rate of positive response to LPS implantation, which was defined as an improvement of one point or more on the NIHSS score within 3 months, was calculated.

### Statistical Analysis

SPSS version 19 (IBM, Armonk, New York) was used to perform statistical analysis. The Kolmogorov–Smirnov test was first used to determine the normality for quantitative data. Continuous data following normal distribution (age, Evans Index, CSF glucose, CSF chlorine) were described as mean ± standard deviation (SD), and *t-*test was used to compare the difference. Other quantitative data (GCS, duration of hydrocephalus, length of stay, CSF nucleated cells, CSF RBCs, CSF protein) were described as median (range), and Wilcoxon rank sum test was used to compare the difference. Categorical data (sex, history of prior shunt, etiology) were described as number (percentage). In the binary logistic regression analysis, first, variables with a *p* < 0.2 in univariate analysis were selected for further multivariate analysis. Probability values (*p*) < 0.05 (two-sided) were considered to have statistical difference.

## Results

### Patients and Characteristics

**Figure 1:**
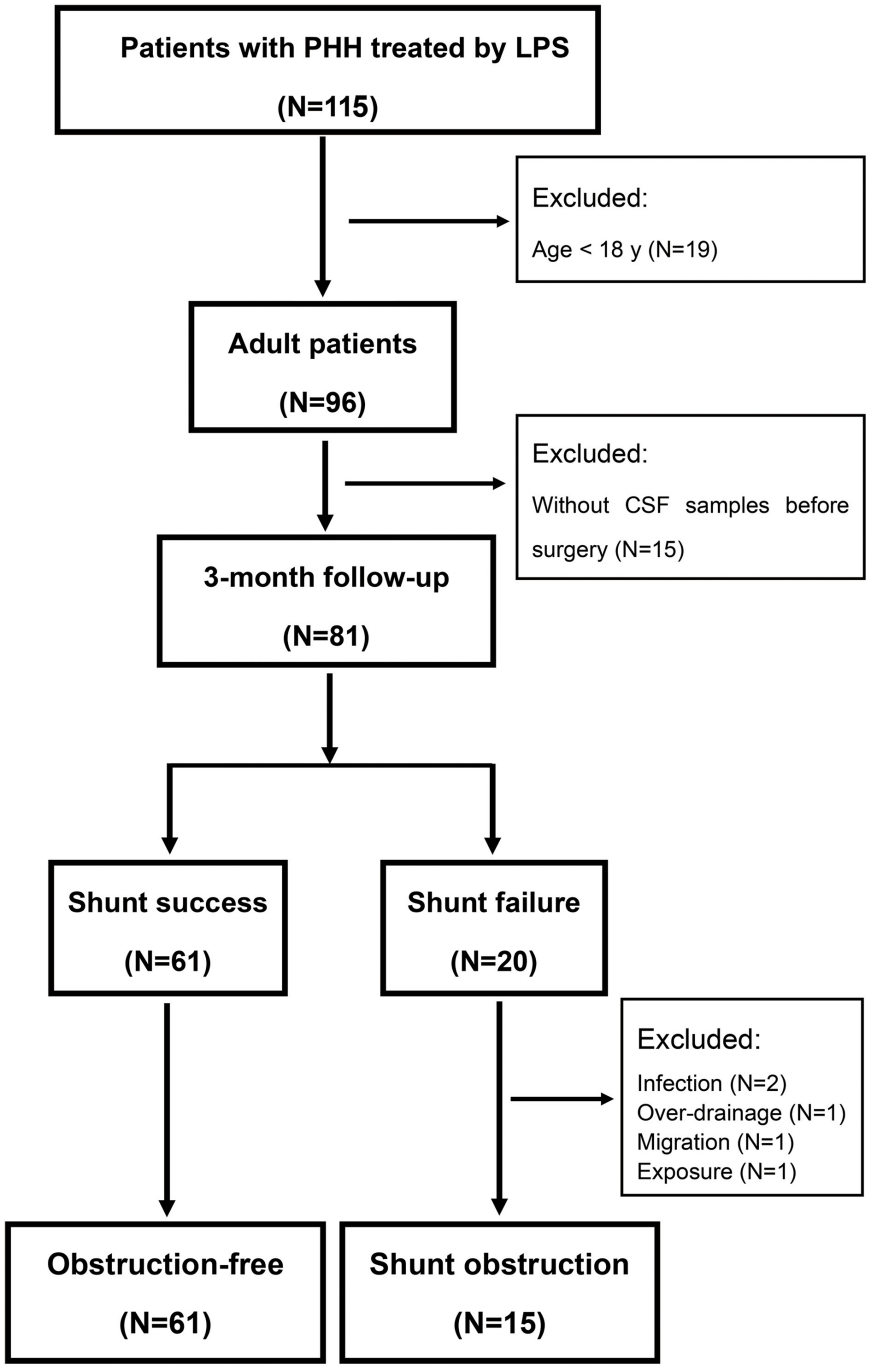
The flowchart of the selection of patients. PHH, post-hemorrhagic hydrocephalus; LPS, lumboperitoneal shunt; CSF, cerebrospinal fluid. Obstruction-free was defined as patients with an improvement of at least one point at NIHSS and without any shunt failure.

The flowchart of the selection of patients is shown in Figure 1. Ninety-six adult patients diagnosed as PHH underwent LPS from years 2014 to 2018 according to the electronic database of the hospital. Fifteen patients without CSF sample examination before shunt insertion were excluded. Among the remaining 81 patients, 61 patients were obstruction-free and 20 patients underwent shunt revisions within 3 months. Specifically, 15 patients were early SO while five patients underwent shunt revisions owing to infection (two patients), over-drainage (one patient), migration (one patient), or exposure (one patient). This study therefore consisted of 76 patients, those who underwent comprehensive CSF analysis within 1 week prior to shunt placement or at the time of shunt implantation (obstruction-free: 61, early SO: 15).

The baseline characteristics are listed in Table 1. Fifty-five were male (72.4%) and 21 were female (27.6%). The mean age at surgery was 49.7 years. Nine patients (11.8%), 39 patients (51.3%), and 10 patients (13.2%) had history of prior shunt, craniectomy, and intracranial infection, respectively. Forty-nine patients (64.5%) were traumatic at onset. The median duration from onset to shunt was 1.0 month (range: 0.1–12), and the median GCS was 11. After shunt surgery, the median length of stay after surgery was 12.5 days (range: 4–139).

**Table 1:** Baseline characteristics.

	**Total (*n* = 76)**	**Control group (*n* = 61)**	**Study group (*n* = 15)**	***p*-value**
Age, y, mean ± SD	49.7 ± 15.8	49.7 ± 16.3	49.6 ± 14.0	0.984
Sex, *n* (%)				0.461
Male	55 (72.4%)	43 (56.6%)	12 (15.8%)	
Female	21 (27.6%)	18(23.7%)	3 (3.9%)	
Etiology, *n* (%)				0.843
Traumatic	49 (64.5%)	39 (51.3%)	10 (13.2%)	
Non-traumatic	27 (35.5%)	22 (28.9%)	5 (6.6%)	
Time from onset to shunt, m, median	1.0 (0.1–12)	1.0 (0.1–12)	1.0 (0.1–3)	0.358
Craniectomy, *n* (%)				0.274
Yes	36 (47.4%)	27 (35.5%)	9 (11.8%)	
No	40 (52.6%)	34 (44.7%)	6 (7.9%)	
History of intracranial infection, *n* (%)				0.382
Yes	10 (13.2%)	7 (9.2%)	3 (3.9%)	
No	66 (86.8%)	54 (71.1%)	12 (15.8%)	
History of prior shunt, *n* (%)				0.489
Yes	9 (11.8%)	8 (10.5%)	1 (1.3%)	
No	67 (88.2%)	53 (69.7%)	14 (18.4%)	
GCS, median (range)	11 (3–15)	11 (3–15)	11 (3–15)	0.683
Type of shunt system, *n* (%)				0.945
Medtronic	31 (%)	25 (%)	6 (%)	
Sophysa	45 (%)	36 (%)	9 (%)	
Length of stay, d, median	12.5 (4–139)	13 (5–139)	20 (4–81)	0.480

In order to identify any factors associated with SO, we synchronously compared the baseline data between these two groups. However, factors highly suspected to be related to SO were not of statistical significance, including age (*p* = 0.981), etiology (*p* = 0.843), history of prior shunt (*p* = 0.489), time from onset to shunt insertion (*p* = 0.358), and type of implanted system (*p* = 0.945).

### Shunting Outcomes

NIHSS dramatically decreased at discharge with statistical significance (median: 12 vs. 9, *p* = 0.032), indicating an immediate improvement of clinical symptoms. In terms of Evans Index, no statistical difference was observed (0.35 ± 0.06 vs. 0.35 ± 0.05, *p* = 0.255) following LPS. The proportion of patients with positive response was 73.7% (56 patients) within 3 months after surgery. The overall rate of early SO was 15.6% (study group, 15 patients) while 61 patients (control group) were obstruction-free without shunt revisions.

### Association of CSF Parameters With Early Shunt Obstruction

The values for CSF parameters and the comparison of the study group to control group are shown in Table 2. RBC count (median: 86 vs. 2, *p* = 0.006) and nucleated cell count (median: 5 vs. 0, *p* = 0.028) of preoperative CSF significantly elevated in the study group compared with control group. However, there was no statistical significance between the two groups in the level of protein (median: 0.48 vs. 0.59, *p* = 315), CSF glucose (3.43 ± 0.92 vs. 3.26 ± 0.89, *p* = 0.510), and CSF chlorine (125.89 ± 9.65 vs. 122.4 ± 6.29, *p* = 0.093).

**Table 2:** Comparison of CSF parameters between two groups.

**CSF parameters**	**Total (*n* = 76)**	**Control group (*n* = 61)**	**Study group (*n* = 15)**	***p*-value**
Protein, g/L	0.5 (0.18–2.77)	0.48 (0.18–2.77)	0.59 (0.25–1.81)	0.315
Nucleated cells, × 10^6^/L	2 (0–486)	0 (0–486)	5 (0–189)	0.028^*^
RBCs, × 10^6^/L	10 (0–20,000)	2 (0–20,000)	86 (0–20,000)	0.006^*^
Glucose, mmol/L	3.29 ± 0.89	3.26 ± 0.89	3.43 ± 0.92	0.510
Chlorine, mmol/L	123.1 ± 7.1	122.4 ± 6.29	125.89 ± 9.65	0.093

*CSF, cerebrospinal fluid*.

* *, with statistical difference*.

In the binary logistic regression analysis as shown in Table 3, variables with *p* < 0.2 in the univariate analysis including elevated CSF nucleated cell counts (>0 × 10^6^/L; OR: 2.842, 95% CI: 0.814–9.915, *p* = 0.101), elevated CSF RBC counts (>0 × 10^6^/L; OR: 13.548, 95% CI: 1.676–109.530, *p* = 0.015), and CSF chlorine level (OR: 1.069, 95% CI: 0.985–1.160, *p* = 0.110) were selected to perform further multivariate analysis. Other CSF parameters were not associated with early SO, including CSF protein level (OR: 1.418, 95% CI: 0.514–3.910, *p* = 0.500) and CSF glucose level (OR: 1.240, 95% CI: 0.659–2.336, *p* = 0.505). Multivariate analysis identified the presence of RBCs in preoperative CSF (>0 × 10^6^/L; OR: 10.629, 95% CI: 1.238–91.224, *p* = 0.031) as a dependent risk factor for early SO while elevated nucleated cells (>0 × 10^6^/L; OR: 1.623, 95% CI: 0.413–6.371, *p* = 0.488) and CSF chlorine level (OR: 1.054, 95% CI: 0.968–1.147, *p* = 0.224) were not of statistical significance.

**Table 3:** Binary logistic regression analysis of the association of CSF parameters with early SO.

	**Univariate analysis**			**Multivariate analysis**		
	**OR**	**95% CI**	***p*-value**	**OR**	**95% CI**	***p*-value**
Protein, g/L	1.418	0.514–3.910	0.500			
Nucleated cells >0 × 10^6^/L	2.842	0.814–9.915	0.101^#^	1.623	0.413–6.371	0.488
RBCs >0 × 10^6^/L	13.548	1.676–109.530	0.015^#^	10.629	1.238–91.224	0.031^*^
Glucose, mmol/L	1.240	0.659–2.336	0.505			
Chlorine, mmol/L	1.069	0.985–1.160	0.110^#^	1.054	0.968–1.147	0.224

# *Variables with p < 0.2 were selected for multivariate analysis*.

* *p < 0.05*.

## Discussion

SO is the most common cause of shunt failure, remaining a significant problem with diversion procedures (12). The incidence of shunt failure ranged from 7 to 85.7% in various studies, and many failures occur during the first year with a continued risk of revision thereafter (5, 8, 9, 11, 15). In our cohort, 20 of 96 patients (20.8%) failed, requiring single or multiple shunt revisions within 3 months after LPS implantation, and the most common reason for revisions was SO (15 patients, 15.6%).

With the advent of CSF shunts, a great deal of attention was directly given to the exploration of path to attenuate the incidence of shunt failure. There is a persistent belief that elevation of CSF parameters, particularly RBC count and protein level, is contributory to early SO, supported by anecdotal and speculative evidence (16). In fact, many surgeons recommended delaying shunt insertion in patients with a high level of CSF parameters. This historical recommendation might appear to be supported by the findings of some early studies that indicated that shunt failure was associated with high protein levels (17). However, there are few experimental studies suggesting that protein did not impair shunt function while RBCs could affect the shunt performance. Brydon et al. (18) perfused the valves with solutions of protein and blood suspensions at varying concentrations, indicating that protein did not affect valve function but that RBC count was associated with shunt failure. Their findings were then demonstrated by the work of Baird et al. *in vitro* (19).

There are also few clinical studies providing speculative evidence. In pediatric cohorts, a high risk of early SO was observed in pediatrics with PHH, compared to those with congenital hydrocephalus or other etiologies (20). However, analysis of CSF samples of low-birth-weight premature infants with PHH treated by VPS did not find any association between shunt failure and RBCs counts, protein level, or glucose level (16). In adult cohorts, Rammos et al. (21) found that elevated level of protein and RBC counts in the CSF did not affect shunt function in patients with hydrocephalus secondary to aneurysmal SAH treated by VPS, which was in line with a subsequent study by Kang et al. (22).

Nevertheless, there are surprisingly no studies examining CSF parameters with SO following LPS implantation in adults. It should be noted that results found in VPS cannot be easily translated to the LPS-treated populations. Given that patients with a high level of CSF parameters before shunt insertion might be at a high risk of SO, we analyzed the RBC count, nucleated cell count, protein level, glucose level, and chlorine level of preoperative CSF and assessed whether CSF parameters were associated with early SO following LPS implantation in a group of adult patients with PHH. The RBC count and nucleated cell count in preoperative CSF were actually higher in patients with early SO. Importantly, patients with RBCs in preoperative CSF (>0 × 10^6^/L) showed a statistically significant increase in the risk of early SO (OR: 10.629, 95% CI: 1.238–91.224, *p* = 0.031), indicating that it might be necessary to wait for blood to clear from the CSF before LPS insertion. Meanwhile, early SO was not statistically associated with CSF protein level, glucose level, and chlorine level. Despite our findings, a further prospective study with a large sample size is essential to draw strong conclusions.

Additionally, LPS was effective and safe in the treatment of PHH according this study. Of 76 patients, 46 (60.5%) showed improvement in NIHSS at the time of discharge while the statistical difference on Evans Index between the two groups was not observed, indicating that the majority of patients obtained favorable outcomes immediately after LPS implantation despite the bare reduction on the ventricular size. Besides, the proportion of patients with positive response (improvement of one point or more on the NIHSS) was 73.7% within 3 months after surgery. Although patients treated by LPS might face some complications, severe adverse events, e.g., subdural hematoma, cerebral infarction, and cerebral hernia, were not observed in the current work (5).

### Limitations

We recognize that the current study still has some limitations. The most critical issue is that the current study is retrospective from a single institution in the presence of possible systematic bias and variation. Second, the evaluation of improvement of symptoms is subjective. There is no scale to assess the symptoms caused by PHH and the commonly used scales describing the severity of INPH, such as INPH grading scale and Keifer's hydrocephalus scale, are not suitable for our cohorts.

## Conclusion

This study suggested that the presence of RBCs in preoperative CSF was associated with an increased risk of early SO in patients with PHH treated by LPS.

## Data Availability Statement

The raw data supporting the conclusions of this article will be made available by the authors, without undue reservation.

## Ethics Statement

Ethical review and approval was not required for the study on human participants in accordance with the local legislation and institutional requirements. The study was conducted according to the guidelines of the Declaration of Helsinki. The patients/participants provided their written informed consent to participate in this study.

## Author Contributions

TS and JG: conceptualization. TS, WC, and SC: formal analysis. TS and XL: methodology. YZ, YY, and HY: investigation. WC and JY: software. CY and JG: funding acquisition and writing—review and editing. JG: project administration. TS and WC: writing—original draft. All authors have agreed to be listed and have seen and approved the manuscript.

## Conflict of Interest

The authors declare that the research was conducted in the absence of any commercial or financial relationships that could be construed as a potential conflict of interest.

## Publisher's Note

All claims expressed in this article are solely those of the authors and do not necessarily represent those of their affiliated organizations, or those of the publisher, the editors and the reviewers. Any product that may be evaluated in this article, or claim that may be made by its manufacturer, is not guaranteed or endorsed by the publisher.
